# Seasonal Changes and Age-Related Effects on the Intestinal Microbiota of Captive Chinese Monals (*Lophophorus lhuysii*)

**DOI:** 10.3390/ani14233418

**Published:** 2024-11-26

**Authors:** Lijing Huang, Yanchu Zheng, Shaohua Feng, Bangyuan Wu, Li Chen, Xiaoqin Xu, Bin Wang, Wanhong Li, Caiquan Zhou, Long Zhang

**Affiliations:** 1 Key Laboratory of Southwest China Wildlife Resources Conservation, Ministry of Education, China West Normal University, Nanchong 637000, China; 2 Sichuan Wildlife Rehabilitation and Breeding Research Center, China West Normal University, Nanchong 637009, China; 3 College of Life Science, China West Normal University, Nanchong 637000, China; 4 Management and Protection Center of Sichuan Fengtongzhai National Nature Reserve, Ya’an 625700, China; 5 Institute of Ecology, China West Normal University, Nanchong 637009, China

**Keywords:** Chinese monal, seasons, ages, gut microbiota, functional prediction, pathogen

## Abstract

The Chinese monal (*Lophophorus lhuysii*) is a large-sized and vulnerable (VU in IUCN) bird from southwestern China. This study applied 16S rRNA high-throughput sequencing to comprehensively examine the gut microbiota of captive Chinese monals (located in Baoxing, Sichuan, China) across varying seasons and life stages. Dominant bacterial phyla identified included Proteobacteria, *Firmicutes*, *Bacteroidetes*, and *Actinobacteria*. Significant seasonal and age-associated shifts were observed within specific bacterial groups, particularly marked by seasonal fluctuations in beta diversity. Moreover, linear discriminant analysis effect size (LEfSe) and functional predictions highlighted distinct winter signatures, indicating possible functional shifts in energy metabolism and disease resistance. In mid-aged adults, an expansion of *Gamma-Proteobacteria* suggested an elevated susceptibility of the gut microbiota of Chinese monals to chronic disorders and microbial imbalance. Putative pathogenic bacteria exhibited increased abundance in spring and summer, likely driven by temperature, host physiological cycles, interspecies interactions, and competition. These findings imply that the diversity, and structure of the gut microbiota in captive Chinese monals are strongly influenced by seasonal and age-related factors. The insights provided here are essential for improving breeding strategies and preventing gastrointestinal diseases in captivity.

## Simple Summary

This study investigated the gut microbiota of captive Chinese monals across different seasons and age groups. The results revealed significant seasonal fluctuations and age-related factors that shape its composition. Specific biomarkers, including *PaeniGlutamicibacter*, *Pseudarthrobacter*, *Micrococcaceae*, and *Micrococcales*, were linked to seasonal changes, while mid-aged adults exhibited a higher abundance of *Kaistella*, *Escherichia*, *Enterobacteriaceae*, and *Enterobacterales*. Furthermore, captive Chinese monals may be more susceptible to bacterial-induced intestinal diseases in spring and summer. This research highlights the dynamic impact of seasonal cycles and age on gut microbiota, offering important findings that support improved conservation strategies and management of captive Chinese monal populations.

## 1. Introduction

The Chinese monal (*Lophophorus lhuysii*, Geoffroy Saint-Hilaire, 1866) [1], a member of the family *Pheasantidae* within the order Galliformes, is a large rare terrestrial bird distributed across southwestern China, including Tibet, Sichuan, Yunnan, Qinghai, and Gansu. Its preferred habitats span alpine meadows, scrub, pine forests, and barren rocky areas at elevations between 3000 and 4900 m [2]. The wild population, currently estimated at 6000 to 10,000 individuals, is experiencing a decline due to limited ecological carrying capacity, human disturbances, and the effects of climate change [3]. The species is classified as globally vulnerable (VU) by the International Union for Conservation of Nature (IUCN), recognized as a Class I nationally protected species in China, and listed in Appendix I of the Convention on International Trade in Endangered Species of Wild Fauna and Flora (CITES) [4,5]. Reproduction and conservation of rare animal species in captivity is recognized as a key strategy for global conservation efforts [6,7]. The artificial breeding and rearing of Chinese monals in captivity have been ongoing since the 1950s. More recently, the 2021 “14th Five-Year Plan for the Protection and Development of Forestry and Grasslands” highlights the imperative to protect the Chinese monal, identifying it as one of the 36 endangered species requiring urgent conservation efforts [8]. In response, captive breeding has been further employed as an approach to establish a foundation for future reintroduction into the wild and aims to secure the species’ long-term viability and reproductive success within its natural ecosystem.

The gut microbiota, a diverse microbial community within the host’s digestive tract, significantly influences host health and physiological functions, including food digestion, nutrient metabolism, and immune system regulation, among other processes [9]. Dysfunction of the microbiota may heighten disease susceptibility and compromise intestinal barrier integrity. Consequently, alterations in the gut microenvironment driven by the microbiota and its metabolic byproducts can profoundly impact regional gut function [10,11]. Host genetic makeup, behavior, dietary patterns, and environmental factors are all key determinants of gut microbiota structure and diversity [12,13,14,15]. Moreover, the microbiota can exhibit adaptive strategies under extreme conditions, such as variations in microbial diversity with altitude in the Eurasian tree sparrow (*Passer montanus*) [16]. Often referred to as the “second genome” of animals, the gut microbiota plays a multifaceted role in supporting host well-being [17]. Disruption of the “microbiome–gut–host” system homeostasis may impair nutritional, metabolic, and immune equilibrium, subsequently triggering chronic inflammatory responses in the host. These sustained inflammatory processes can contribute to the onset of various pathologies, ultimately compromising animal health [18]. While captive conservation provides essential resources for species survival, it also poses greater risks than natural habitats, risks closely tied to the host’s gut microbiota composition [19]. Consequently, conservation efforts must prioritize not only the protection of species’ genetic diversity but also the preservation of the gut microbiota’s diversity and specificity within the species, thereby maintaining ecological balance and long-term health stability.

Building on the growing insights into gut microbiota, these communities are now recognized as dynamic, influenced by both environmental factors such as temperature and food availability, and physiological conditions, resulting in adaptive adjustments to their structure [20,21]. Diet remains a primary determinant shaping the composition and diversity of gut microbiota, with investigations spanning multiple species, including grass carp [22], Tibetan macaques [23], giant pandas [24], and red-crowned cranes [25]. Temporal factors, including the host’s age and seasonal environmental fluctuations, also modulate gut microbiota composition and diversity [26,27]. As hosts age, the physiological changes and evolving nutritional requirements, coupled with varying absorption capacities, contribute to alterations in microbial communities. Notable shifts in gut microbiota composition have been observed across different growth stages in species such as chickens, sparrows, and crested ibis [28,29,30]. Seasonal and age-related factors have significant impacts on gut microbiota dynamics in both captive and wild animals, such as partridges [31], pigeons [32], ducks [33], common kestrels [34], catharus thrushes [35], and shorebirds [36]. Seasonal shifts predominantly influence gut microbiota structure and composition by modifying diet, environmental exposure, and behavioral patterns, thereby affecting microbiota diversity and function. At different life stages, the host’s gut microbiota is influenced by a range of factors, necessitating distinct protective measures and management strategies to preserve microbiota stability and functionality, which is vital for the overall health of the host population. Furthermore, the structural composition of gut microbiota can act as a reliable biomarker in assessing whether captive animals have achieved the physiological conditions required for successful wild reintroduction.

Given its significance, understanding the composition, functional differences, and presence of potential pathogens in the gut microbiota of captive Chinese monals is crucial for improving their health status. To this end, 16S rRNA Sequencing was conducted in this study to assess the impact of seasonal environmental fluctuations and host age on the composition of gut microbiota. The results not only provide baseline data for the effective management and conservation of this endangered species but also enhance our understanding of the gut microbiota in captive Chinese monals and offer practical insights for ex-situ conservation efforts.

## 2. Materials and Methods

### 2.1. Sample Collection

Under consistent captive conditions, this study collected 44 fecal samples from captive Chinese monals in January (winter), April (spring), July (summer), and October (autumn) of 2022 at the Sichuan Baoxing Fengtongzhai Nature Reserve Management Center (altitude: 2000 m; longitude: 29°11′12.74″ N, 102°09′36.15″ E; captive breeding approved in 1992). The samples were evenly distributed by season, with 11 samples collected each season. The reserve contains 11 enclosures, housing 1–2 monals each (Supplementary Figure S1). The daily diet of the captive Chinese monals consisted primarily of corn, carrots, cabbage, cooked eggs, tomatoes, mealworms, and peanuts. Water and food were provided ad libitum and refreshed daily. Clean cardboard was used in the nighttime roosting areas, and 3–4 fecal samples were collected from each enclosure the following morning. Given the inability to trace each fecal sample to a specific individual, samples from each enclosure were pooled during collection. Subsequently, approximately 3 g of fecal matter from the center of each mixed sample was transferred into sterile 15 mL centrifuge tubes and stored at −80 °C for later DNA extraction. In the captive population, Chinese monals begin producing fertilized eggs as early as age 3, and both males and females retain reproductive capacity until at least age 18 (with an estimated lifespan of 25.5 years) [37,38]. Based on age, the samples were divided into two groups: mid-aged adults (10–18 years) and young adults (3–5 years). After excluding 9 samples that did not meet the criteria—due to either falling outside the age range or containing mixed-age individuals in the same enclosure—33 samples remained, with 15 from the older group and 18 from the younger. It should be noted that, as the sampling times were limited to specific months (January, April, July, and October), it was challenging to fully observe the dynamic changes in the microbiome across each season. Additionally, individual samples were not differentiated during sampling, making it impossible to analyze differences between males and females. We will take these methodological limitations into account when interpreting the results.

### 2.2. DNA Extraction and 16S rRNA Sequencing Processing

Fecal microbial genome DNA was extracted using the fecal microbiota genomic DNA extraction kit (DP328, Beijing Tiangen Biochemical Technology Co., Ltd., Beijing, China), following the manufacturer’s protocol. DNA quality was evaluated via 1.5% agarose gel electrophoresis, and concentration was quantified using a Thermo NanoDrop 2000 UV spectrophotometer (Thermo Fisher Scientific, Waltham, MA, USA). Only DNA samples meeting quality standards were processed further. Amplification of the 16S rRNA V3-V4 region was performed with universal bacterial primers (forward: 5′-AGAGTTTGATCCTGGCTCAG-3′, reverse: 5′-GGTTACCTTGTTACGACT-3′). The PCR conditions included an initial denaturation at 98 °C for 2 min, followed by 35 cycles of denaturation at 98 °C for 30 s, annealing at 55 °C for 30 s, and extension at 72 °C for 30 s, with a final extension at 72 °C for 5 min. Polymerase chain reaction (PCR) products were held at 10 °C until removal. Purified and quantified PCR products were then used to construct sequencing libraries. Sequencing was conducted on the IlluminaMiSeq platform (Illumina, San Diego, CA, USA) by Shanghai Biozeron Biotechnology Co., Ltd. (Shanghai, China).

### 2.3. Biological Information Analysis

The raw sequencing data from all samples were processed using QIIME2 software (version 2021.2, https://qiime2.org, accessed on 23 November 2023) [39] for the removal of adapters and barcodes. Subsequent quality control and filtering steps were applied to generate high-quality clean data, with chimeric amplicons eliminated via VSEARCH [40]. Sequences were clustered according to the 100% similarity criterion of amplicon sequence variants (ASVs). Species identification was conducted on representative sequences of each ASV using the SILVA and RDP reference databases (version 138.1, version 11.4) [41]. Microbial composition analyses were performed at various taxonomic levels, excluding ASVs with relative abundance below 0.01%. The relative abundance was calculated using the following formula: relative abundance (%) = [(number of each ASV across all samples/total number of ASVs across all samples) × 100].

### 2.4. Analysis of Potential Pathogen

A comprehensive manual search was performed using identified species data in combination with keywords like “enteric pathogens”, “pathogenic bacteria”, “gut opportunistic pathogens”, and “potential intestinal pathogens” across platforms such as Google Scholar, Web of Science, and PubMed. Drawing from existing literature and LPSN (https://lpsn.dsmz.de/, accessed on 23 July 2024), 25 potentially pathogenic bacterial species were identified for further analysis. These species have been validated as pathogens associated with disease in both humans and animals.

### 2.5. Data Processing and Analysis

Data visualization was performed using the bioanalysis platform of LC-Bio (Lianchuan Biotechnology Co., Ltd., Hangzhou, China, https://www.omicstudio.cn/tool, accessed on 7 June 2024). A Venn diagram generated from the ASVs illustrates the unique and shared microbiota between the groups, while a stacked bar plot highlights the top 20 most abundant microbiota to visualize their interactions with the different groups. Linear discriminant analysis effect size (LEfSe) was applied to identify inter-group variations in microbial communities, represented through a branching diagram for groups exhibiting differential abundance (LDA score > 4.0, *p* < 0.05). The overall biological heterogeneity of a given environmental community, or diversity, was assessed through alpha and beta diversity metrics. Alpha diversity, measured via four indices (Chao1, Shannon, Simpson, Ace), provided insights into species richness and evenness within the gut microbiota of the Chinese monal. Beta diversity, visualized through Non-metric Multidimensional Scaling (NMDS) and Principal Coordinate Analysis (PCoA), revealed variations in the composition of gut bacterial communities across different seasons and age groups. To explore microbial community interactions, the Sparse Correlations for Compositional Data (SparCC) algorithm was employed, detecting both positive and negative correlations among taxa. Potential pathogens (*n* = 25) identified from the relative abundance data were Log10-transformed, with a heatmap used to visualize their distribution within the gut microbiota of Chinese monals by season and age. Finally, ASV species annotation and abundance data were integrated with the Kyoto Encyclopedia of Genes and Genomes (KEGG) database, while metabolic functional predictions of the gut bacterial community were performed using the Phylogenetic Investigation of Communities by Reconstruction of Unobserved States (PICRUSt2, version 2.4.0) software [42].

## 3. Results

### 3.1. Effective Data Statistics of Samples

**Figure 1:**
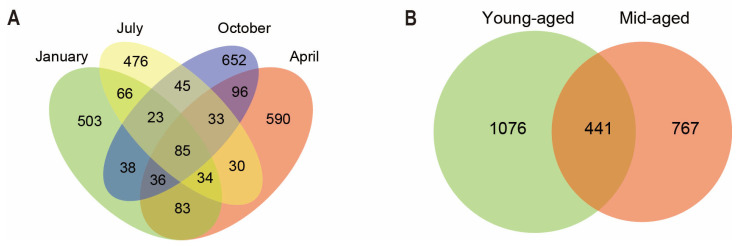
Distribution of ASV in fecal samples of Chinese monal in different seasons (**A**) and ages (**B**).

Through data processing, 1,576,450 high-quality sequences were generated from 44 fecal samples of Chinese monals, with sequence lengths spanning 1400–1500 bp, resulting in the clustering of 2790 bacterial ASVs. The highest number of unique ASVs was detected during autumn, while summer exhibited the lowest. As illustrated in Figure 1A, 85 sequences were common across the four seasons, with overlapping ASVs ranging from 23 to 96. Additionally, 441 ASVs were shared between individuals at the two age stages, while 1076 and 767 unique ASVs were identified in young-aged and mid-aged adults, respectively (Figure 1B).

### 3.2. Structure and Composition of Gut Microbiota in Chinese Monal of Different Seasons and Ages

**Figure 2:**
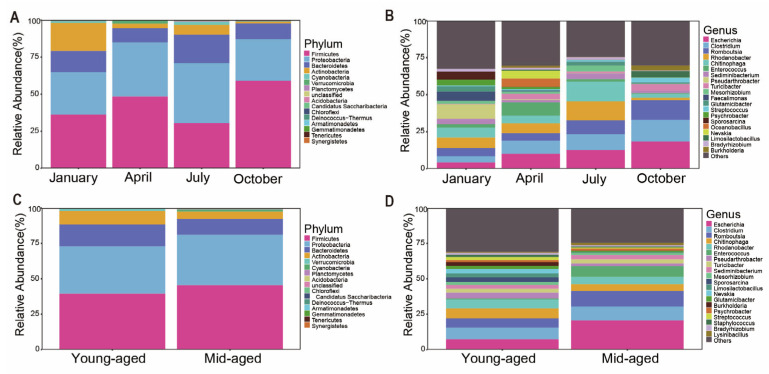
The relative abundance of gut microbiota in captive Chinese monals across different seasons (**A**,**B**) and age groups (**C**,**D**) was presented at both the phylum and genus levels. Panels (**A**,**C**) illustrate microbial composition at the phylum level, while panels (**B**,**D**) depict the distribution at the genus level.

With the aforementioned strategy, sequences from the four-month samples were categorized into 16 phyla and 409 genera, while those from two age groups were assigned to 16 phyla and 361 genera. The predominant bacterial phyla across both groups were *Firmicutes*, *Proteobacteria*, *Bacteroidetes*, and *Actinobacteria*, collectively representing over 97% of the total composition (Figure 2 and Supplementary Figures S2 and S3). Among the seasons, *Firmicutes* reached peak relative abundance in October (59.0%) and was least abundant in July (30.5%). *Proteobacteria* showed the highest proportion in July (40.5%) and the lowest in October (28.2%). *Bacteroidetes* peaked in July (19.2%) and had its lowest presence in April (9.7%). *Actinobacteria* displayed its highest proportion in January (18.9%) and the lowest in October (1.3%) (Figure 2A and Supplementary Table S1). Mid-aged adults exhibited higher levels of *Firmicutes* and *Proteobacteria*, with proportions of 45.6% and 35.7%, respectively, while younger individuals had slightly elevated levels of *Bacteroidetes* (15.4%) and *Actinobacteria* (9.7%) (Figure 2C). At the genus level, the dominant bacterial genera in captive Chinese monals included *Escherichia*, *Clostridium*, *Romboutsia*, *Chitinophaga*, *Enterococcus*, and *Rhodanobacter*. Certain genera showed marked differences in abundance. For example, *Burkholderia* was most abundant in October (3.3%) but significantly lower in January and July (0.1%) (Figure 2B and Supplementary Table S1). *Escherichia* was nearly twice as abundant in mid-aged adults compared to younger individuals (Figure 2D and Supplementary Table S1).

**Figure 3:**
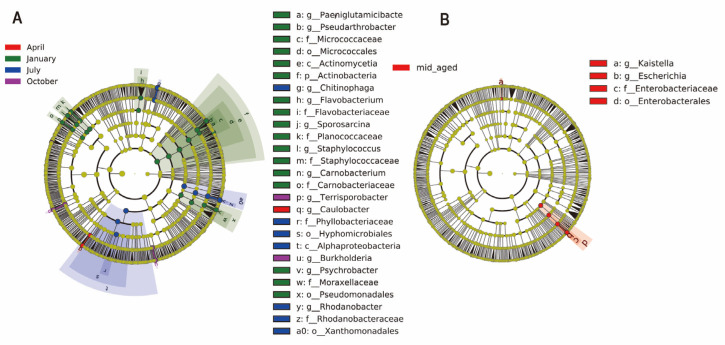
Seasonal and age-related variations in gut microbiota abundance in Chinese monals were assessed. LEfSe analysis, incorporating the Kruskal-Wallis test (*p* < 0.05) and an LDA score threshold of 4.0, was employed to detect significant microbial differences across groups. A cladogram illustrates the seasonal shifts in enriched bacterial taxa (**A**), while a separate cladogram highlights age-related differences in microbial abundance (**B**). The letters preceding ASVs denote taxonomic ranks: p = phylum, c = class, o = order, f = family, g = genus, s = species.

LEfSe analysis was applied to examine taxonomic shifts in the gut bacterial composition of captive Chinese monals, spanning from phylum to species levels across four seasons and two age stages. A total of 42 bacterial taxa displayed LDA scores exceeding 4. Notably, April showed only two distinct bacterial communities, while January revealed 24 differential taxa, a significantly higher count compared to other months (*p* < 0.05, Figure 3A & Supplementary Figure S4A). Winter microbiota exhibited significant variability, encompassing four phyla, four classes, five orders, six families, eight genera, and seven species, including *PaeniGlutamicibacter, Pseudarthrobacter, Micrococcaceae, Micrococcales, Actinomycetia*, *Actinobacteria*, *Flavobacterium, Flavobacteriaceae, Sporosarcina, Planococcaceae, Staphylococcus, Staphylococcaceae, Carnobacterium, Carnobacteriaceae, Psychrobacter, Moraxellaceae*, and *Pseudomonadales*, primarily from *Proteobacteria*, *Firmicutes*, and *Actinobacteria*. Similarly, six bacterial taxa differentiated between young and mid-aged adults, including *Kaistella, Escherichia, Enterobacteriaceae*, *Enterobacterales*, *Escherichia flexneri*, and *Escherichia dysenteriae*. The bacterial diversity in mid-aged adults was significantly higher than in younger individuals (Figure 3B & Supplementary Figure S4B).

### 3.3. Alpha and Beta Diversity of Captive Chinese Monals Across Seasons and Ages

**Figure 4:**
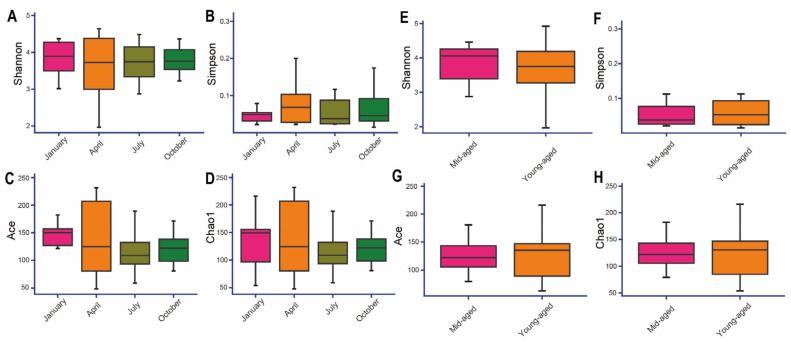
Seasonal and age-related variations in the gut microbiota of the Chinese monal were assessed through α diversity metrics. Panels (**A**,**E**) display the Shannon index, (**B**,**F**) represent the Simpson index, (**C**,**G**) illustrate the Ace index, while (**D**,**H**) depict the Chao1 index.

The Goods coverage index for all samples surpassed 0.99, affirming the accuracy and dependability of the sequencing data (Supplementary Table S2). The Shannon and Simpson indices indicated the highest species diversity in January, followed by October, July, and April (Figure 4A,B). In contrast, the Chao1 and Ace indices revealed that species richness peaked in July and was lowest in April (Figure 4C,D). When comparing age groups, the young-aged cohort exhibited higher Chao1 and Ace indices but lower Shannon and Simpson values, suggesting increased gut richness but diminished microbial diversity relative to the mid-aged group (Figure 4E–H). Despite these variations, none of the alpha diversity indices showed statistically significant differences across the analyses (*p* > 0.05). This indicates that although there are trends in species richness and diversity across different seasons and age groups, these differences may be attributed to random factors or environmental influences, rather than reflecting a significant biological impact.

**Figure 5:**
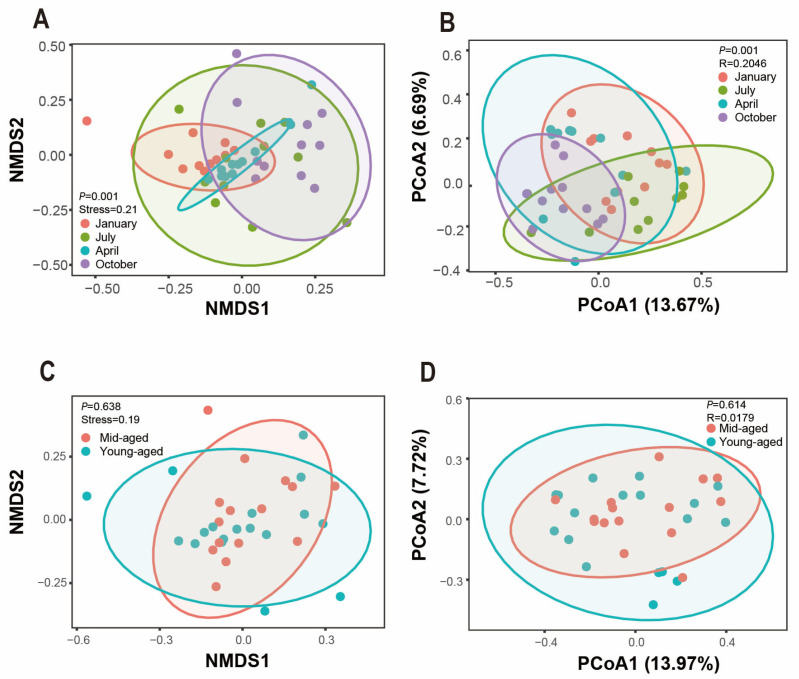
The beta diversity of the gut microbiota composition in Chinese monals was assessed across different seasons (**A**,**B**) and age groups (**C**,**D**). NMDS and PCoA were employed to evaluate the variations in gut microbiota communities, with statistical significance denoted by *p* values (*p* < 0.05). Each color corresponds to a distinct group, where proximity between samples indicates greater similarity in microbial composition and structure, while greater distance signifies increased dissimilarity. Panels (**A**,**C**) display the results from NMDS and panels (**B**,**D**) from PCoA.

To investigate inter-community variation, NMDS and PCoA analyses were employed to assess beta diversity across the groups. Significant differences in Bray–Curtis distances were identified among the four seasonal time points (*p* < 0.05), indicating distinct clustering of bacterial communities by season and revealing the unique composition of gut microbiota in captive Chinese monals across seasons (Figure 5A,B). Conversely, no significant variation in beta diversity was detected between young and mid-aged adults (*p* > 0.05). While some degree of clustering was observed among certain samples, changes in gut microbiota with age appeared limited (Figure 5C,D).

### 3.4. Functional Predictions

**Figure 6:**
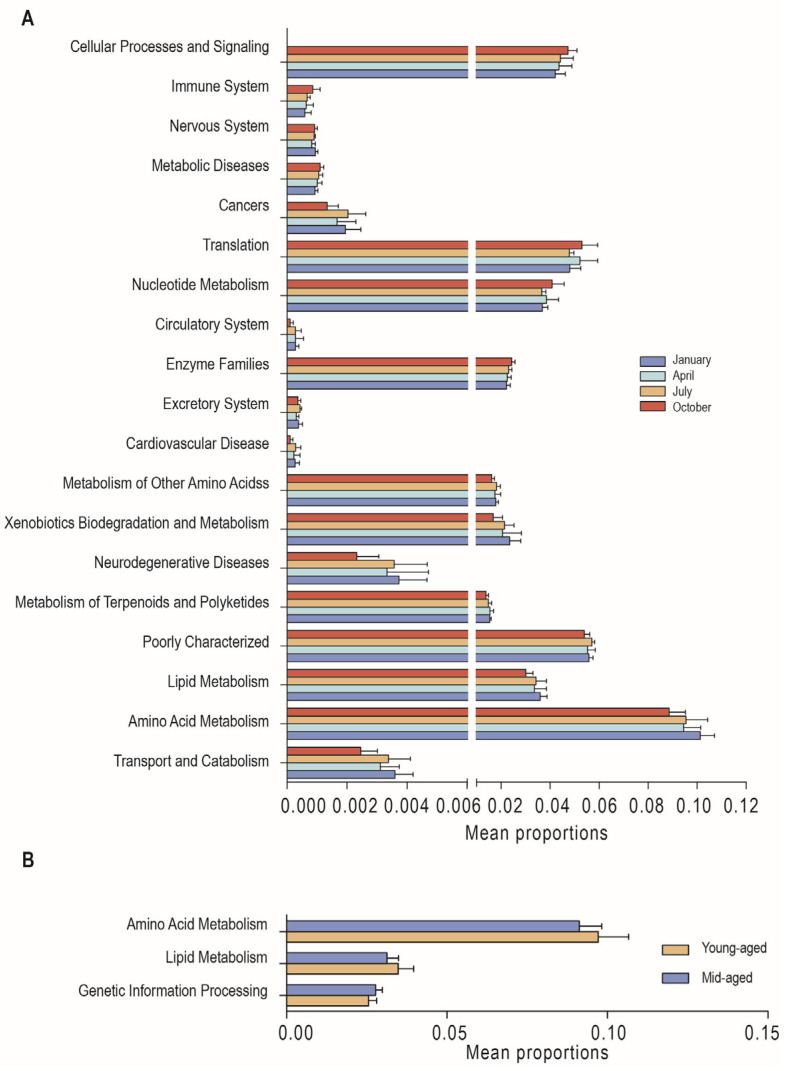
Differences in KEGG metabolic pathways of Chinese monal in different seasons (**A**) and ages (**B**).

Using PICRUSt2 function predictions, KEGG metabolic pathways were analyzed to assess the functional diversity of gut microbiota across various comparisons. For secondary KEGG pathways, 19 distinct functional pathways were identified across the four seasons, along with 123 differential tertiary pathways (Supplementary Tables S3 and S4). In January, enrichment was notably higher in pathways related to transport and catabolism, amino acid metabolism, lipid metabolism, neurodegenerative diseases, xenobiotic biodegradation, and metabolism. Significant enrichment in April and July was observed for the metabolism of terpenoids and polyketides and for the metabolism of other amino acids, respectively. In October, differential microbial abundances were associated with enzyme families, nucleotide metabolism, and translation compared to other months (Figure 6A). Additionally, three secondary and 19 tertiary differential pathways were identified between individuals at different age stages (Supplementary Tables S5 and S6). Young-aged adults exhibited significantly higher levels of amino acid metabolism and lipid metabolism, while mid-aged adults showed greater enrichment in genetic information processing (Figure 6B).

### 3.5. The Relationship Among Microbial Communities

**Figure 7:**
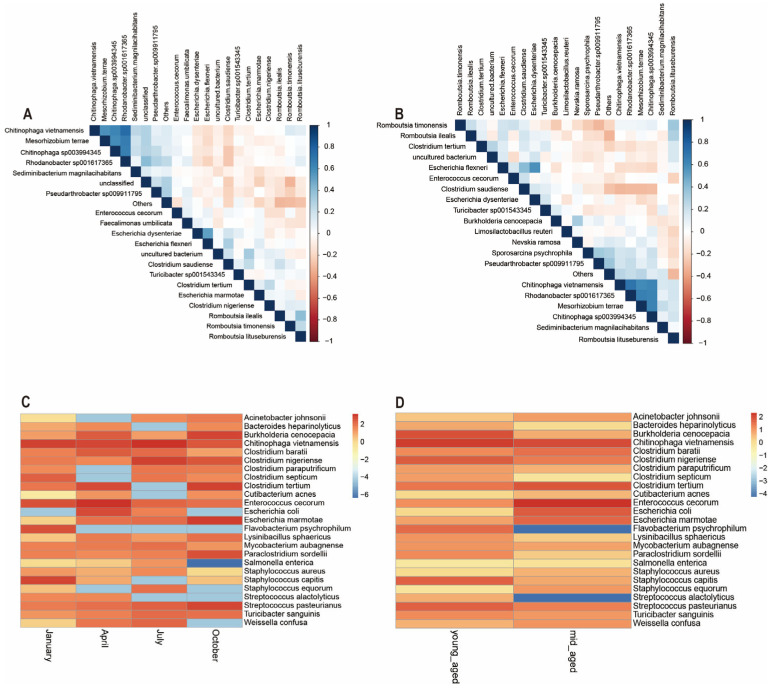
Seasonal (**A**,**C**) and age-related (**B**,**D**) variations in the SparCC heatmaps and relative abundance of potential pathogenic bacteria in the gut microbiota of Chinese monals were highlighted.

Microbial abundance at the species level for the top 20 species was evaluated by analyzing correlations and significance *p* values between dominant bacterial communities, followed by the generation of a correlation heatmap. As depicted in Figure 7A, *Enterococcus cecorum*, *Chitinophaga vietnamensis*, *Clostridium tertium*, *Clostridium nigeriense*, and *Escherichia marmotae* exhibit negative correlations. Similarly, *Romboutsia timonensis*, *Chitinophaga sp003994345*, *Sediminibacterium magnilacihabitans*, *Turicibacter sp001543345*, and *Mesohizobium terrace* were largely negatively correlated with one another. These two groups of bacterial species, however, show a positive correlation between the groups themselves. The relationships among gut microbiota species in Chinese monals, when stratified by age, mirror those observed across different seasons, though certain species correlations were more pronounced in the age group comparisons. For example, the negative correlations between *Chitinophaga vietnamensis*, *Enterococcus cecorum*, and *Clostridium tertium* were more distinct (Figure 7B). Additionally, based on literature review and abundance data, 25 potential pathogenic bacteria were identified. A heatmap was employed to examine the distribution of potential pathogenic bacteria’s relative abundance in the gut of Chinese monals across different seasons and age groups. The analysis revealed that the relative abundance of key potential pathogens was elevated in spring and summer, with lower levels observed during autumn and winter. Notably, certain pathogens exhibited distinct seasonal patterns: *Burkholderia cenocepacia*, *Flavobacterium psychrophilum*, *ParaClostridium sordellii*, and *Streptococcus pasteurianus* were more prevalent in autumn and winter, whereas *Salmonella enterica* was nearly absent during these periods (Figure 7C). Regarding age, most potential pathogens showed no consistent trends, with fluctuations within the expected range. However, the relative abundance of *Flavobacterium psychrophilum* and *Streptococcus alactolyticus* was significantly higher in mid-aged adults compared to younger individuals (Figure 7D).

## 4. Discussion

Through non-invasive fecal sampling and 16S rRNA sequencing technology, we conducted a comprehensive study on the gut microbiota composition, diversity, metabolic functions, and potential pathogens in captive Chinese monals across seasons and at different life stages. The results show that the gut microbiota composition of captive Chinese monals predominantly consists of *Proteobacteria*, *Firmicutes*, *Bacteroidetes*, and *Actinobacteria* at the phylum level (Figure 2, Figures S1 and S2), aligning with previous reports on avian gut microbiota [43,44]. *Firmicutes* are critical for enhancing cellulose degradation by microorganisms in the host digestive tract [45], while *Bacteroidetes* are integral to the host’s breakdown of oligosaccharides [46]. The observed high abundance of *Firmicutes* and relatively lower proportion of *Bacteroidetes* in captive Chinese monals can likely be attributed to a diet rich in corn and protein sources, such as cooked eggs, mealworms, and peanuts, which mirrors patterns seen in captive red-crowned cranes and vultures [25,47]. In contrast, the gut microbiota of mammals is more influenced by a high-fiber diet [23,24].

Environmental temperature and host physiological cycles are recognized as key factors influencing captive animals with relatively stable diets [29,48,49]. Shifts in temperature are widely acknowledged to significantly impact host adaptability, including nutrient assimilation, microbiota colonization, and immune regulation [50]. On the one hand, temperature can taxa-specifically affect microbial growth, and therefore directly impact the abundance and metabolic pathways of host gut microbiota. On the other hand, it can indirectly regulate microbiota composition by influencing host stress and immune responses. SparCC analysis uncovers a complex interaction network among microorganisms at the species level, revealing potential “cooperative” or “competitive” relationships between different taxa [34]. *Enterococcus cecorum* and *Escherichia marmotae* are classified under the genus *Escherichia*, while *Clostridium tertium* and *Clostridium nigeriense* belong to the genus *Clostridium*, with *Romboutsia timonensis* categorized under the genus *Romboutsia*. *Escherichia* exhibits a negative correlation with *Clostridium* but a positive correlation with *Romboutsia*. Additionally, *Escherichia* shows positive correlations with other genera such as *Clostridium* and *Streptococcus*, while displaying negative correlations with *Glutamicibacter* and *Chitinophaga* (Figure 7A). These interactions reflect the intricate relationships within the gut microbiota, which are closely connected to the host’s nutritional metabolism and immune modulation, while also indicating a self-regulating dynamic equilibrium essential for maintaining gut microbiota stability in captive Chinese monals [51,52,53,54].

In line with previous reports [33,50,55], a reduced relative abundance of *Bacteroidetes* alongside an increase in *Firmicutes* was detected in Chinese monals during October (Figure 2A). Additionally, a significant enrichment of enzyme families, as well as nucleotide metabolism and translation-related pathways during this period, suggests enhanced structural integrity and cell division activity within the gut (Figure 6A and Supplementary Table S3). Such alterations likely support the species’ adaptation to heightened energy demands in response to colder temperatures. Interestingly, this seasonal pattern was confined to October in Chinese monals, whereas partridges and pigeons exhibit a similar trend in winter [31,32], and captive ducks show similar changes during winter and spring [33]. This divergence may result from species-specific energy metabolism strategies, although the possibility that Chinese monals, residing at high altitudes, initiate gut microbiome adjustments earlier to cope with cold environments cannot be ruled out [16,49,56]. Recent research has revealed a range of functions within *Burkholderia* species, with some associated with lung infections [57,58], while certain species are known to produce various antibiotics [59]. A higher relative abundance of *Burkholderia* was detected in autumn (Figure 2B), potentially creating conditions more favorable for infections or influencing the health of captive Chinese monals through microbiome regulation via antibiotic secretion. *Actinobacteria*, known for decomposing organic matter and nutrient cycling in ecosystems [59,60], are also prominent antibiotic producers [61,62]. Although direct evidence linking Actinomycetes to avian health is lacking [21], further investigation is required to elucidate the specific roles of avian *Actinomycetes* and to assess whether *Burkholderia* and *Actinobacteria* may synergistically affect bird growth and health through their antibiotic production. Consistent with previous research [33,63], the results indicated greater microbial divergence during autumn and winter (Figure 4A–D). This pattern implies that under colder conditions, the gut microbiota of captive Chinese monals may undergo changes driven by environmental pressures, leading to an increase in gut microbiota diversity. This could enhance functional diversity and redundancy, optimize energy utilization, and potentially improve disease resistance [19]. PICRUSt analysis revealed significant enrichment of functional pathways across seasons, with 19 pathways showing seasonal variation (Figure 6A & Supplementary Table S3). In January, the neurodegenerative disease pathway, which responded to climate and environmental fluctuations, was notably enriched [64]. Additionally, elevated functions in amino acid and lipid metabolism likely reflected increased energy and neurotransmitter demands during winter, thereby enhancing energy metabolism and maintaining immune defense [65,66]. These findings suggest that seasonal microbiota variations are closely tied to energy metabolism, and tailored, season-specific diets may support better adaptation of monals to captivity.

The relative abundance of *Escherichia* in mid-aged adults was double that observed in young-aged adults (Figure 2D). Current knowledge suggests that *Escherichia* efficiently utilizes nutrients, including carbohydrates and short-chain fatty acids, within the host’s digestive system [67,68]. Functional predictions revealed significant enrichment in lipid and amino acid metabolism pathways in the young-aged group (Figure 6B and Supplementary Table S5), which are integral to cell membrane production, energy storage, and the generation of signaling molecules [69]. This enrichment in younger birds suggests a heightened metabolic state, supplying substantial energy and biosynthetic materials to support growth and activity [70,71]. In contrast, the increased abundance of *Escherichia* in mid-aged birds likely correlates with shifts in physiological metabolic demands across different life stages [72,73]. Moreover, the enhanced reliance on microorganisms for energy utilization is potentially linked to the decline in host metabolic rates with aging [74,75]. As the host ages, immune system maturation potentially enhances immune tolerance, facilitating the establishment of a symbiotic relationship with gut microbiota [76]. Consequently, microbial diversity tends to increase with age, reaching relative stability in adulthood. However, aging also impacts gastrointestinal function, leading to alterations in gut microbiota composition and function, which may result in reduced microbial abundance and diversity [77,78]. In the case of captive Chinese monals, both alpha and beta diversity showed no significant variation between the two age groups (Figure 5C,D), which is consistent with previous studies [34,35]. This suggests that overall gut microbiota structure undergoes minimal changes from sexual maturity to post-reproductive age. However, Figure 1B shows that only 18% of ASVs are shared between the two age groups, indicating that although the overall diversity remains stable, there may be significant differences in the specific taxonomic units that make up this diversity. Phylogenetically, *Proteobacteria* can be further classified into six subgroups: alpha-, beta-, gamma-, delta-, theta-, and epsilon-*Proteobacteria* [79]. An elevated abundance of *Proteobacteria*, predominantly composed of pro-inflammatory bacteria, is frequently linked to disorders and dysbiosis. In particular, the expansion of *gamma-Proteobacteria* is regarded as a key contributor to age-related dysbiosis, creating a feed-forward loop of intestinal degradation and inflammation [80,81,82]. LEfSe analysis revealed a significant increase in *Gamma-Proteobacteria* in mid-aged adults (Figure 3B and Figure S3B), indicating a strong correlation with intestinal barrier dysfunction and chronic inflammation in this group. Additionally, the significant enrichment of pathways related to genetic information processing in the mid-aged group suggests a possible association with gut microbiota repair mechanisms, potentially mitigating age-related intestinal disorders and dysbiosis by maintaining cellular functions and repairing DNA damage [83,84]. Consequently, probiotic administration emerges as a potential strategy to regulate intestinal function and mitigate the progression of age-related dysbiosis in mid-aged individuals. It is worth mentioning that, due to the uneven distribution of microbiota throughout the gastrointestinal tract, fecal sampling cannot fully represent all microbial species within the host’s gut. However, as the optimal choice under conservation constraints, fecal sampling still provides meaningful results for the gut microbiota of captive Chinese monals across different seasons and age groups in this study.

Pathogen analysis allows for the identification of harmful microorganisms in the environment and the assessment of disease transmission risks to hosts [57,85,86]. Consistent with earlier studies [36,87,88,89], the relative abundance of most potential pathogens increased during spring and summer (Figure 7C), suggesting that reproductive cycles and elevated temperatures may enhance the proliferation, transmission, and pathogenicity of certain pathogens. Notably, the relative abundance of Salmonella enterica declined in autumn, in contrast to the trends observed for *Burkholderia cenocepacia* and *Flavobacterium psychrophilum*. These results likely come from competition in resources and space within similar ecological niches [90,91]. For example, their abundance may be affected by the aforementioned secretion of antimicrobial compounds from specific microorganisms. Additionally, such patterns may partially reflect complex interactions between the microbiota and the host [92]. Gram-negative bacteria, such as *Clostridium*, utilize quorum sensing signals to regulate not only microbial community dynamics but also the physiological states of host cells. The autumnal enrichment of *Clostridium* (Figure 2B) may influence gut microbiota composition, suppress inflammatory responses, and reinforce intestinal barrier integrity by secreting antimicrobial compounds [93,94,95,96]. While the pathogens examined in this study were classified at the species level, definitive pathogenicity could not be established for all species. For instance, *Clostridium tertium*, *Burkholderia cenocepacia*, *Streptococcus alactolyticus*, and *ParaClostridium sordellii* are recognized as opportunistic pathogens, exhibiting pathogenicity only under certain conditions, such as microenvironmental shifts or compromised host immunity [97,98,99,100]. Based on this finding, it is essential to strengthen health monitoring and disease control measures for captive Chinese monals, particularly during the spring and summer. This can be achieved through the judicious use of antibiotics when necessary, as well as the administration of probiotics. Additionally, improving temperature and humidity control within the captive environment is vital to minimizing the risk of pathogen transmission. Special attention should be given to the reproductive health of Chinese monals in April (breeding season) to prevent disease outbreaks caused by imbalances in the gut microbiota. In the future, the integration of metagenomic and metabolomic approaches may be necessary to better delineate the specific roles and metabolites of gut microbiota and reduce infection risks associated with these pathogens. These measures will help improve the survival and long-term health management of captive Chinese monals.

## 5. Conclusions

In summary, this study demonstrated that the gut microbiota of captive Chinese monals exhibited clear seasonal variations in beta diversity, while age-related changes in both alpha and beta diversity were comparatively less significant. Seasonal shifts in gut microbiota composition were primarily associated with lipid and amino acid metabolism, likely reflecting temperature-driven adaptations in energy requirements and disease prevention within the captive environment. An increased proportion of *Proteobacteria* in mid-aged monals suggested the onset of age-related intestinal inflammation and compromised intestinal barrier function. Additionally, a higher relative abundance of potential pathogens during spring and summer appeared influenced by interspecific interactions and competition. This study provides valuable insights for developing management strategies to prevent intestinal disorders and offers essential guidance for improving the management of captive populations prior to reintroduction into the wild.

## Data Availability

All data in this study are available upon request by contact with the corresponding author. The datasets generated in this study are accessible through the NCBI Sequence Read Archive under BioProject PRJNA1164957 with the accession number SUB14748306 (https://dataview.ncbi.nlm.nih.gov/object/PRJNA1164957?reviewer=a35r67fij3u2uursdn577lmr83, accessed on 25 September 2024).

## Supplementary Materials

The following supporting information can be downloaded at: https://www.mdpi.com/article/10.3390/ani14233418/s1, Figure S1: Individual information of captive Chinese monals. Figure S2: Characteristics of the gut microbiota structure of the Chinese monal in different seasons. Figure S3: Characteristics of the gut microbiota structure of the Chinese monal in different ages. Figure S4: Significant taxonomic differences in the gut microbiota of Chinese monals between different seasons (A) and age groups (B) based on LDA scores. Table S1: The relative abundance table of the gut microbiota of captive Chinese monals across different seasons and age groups. Table S2: Partial indices for alpha diversity analysis of Chinese monal in dfifferent seasons and ages. Table S3: Predicted functions of KEGG level 2 pathways for the Chinese monal in different seasons. Table S4: Predicted functions of KEGG level 2 pathways for the Chinese monal at different ages. Table S5: Predicted functions of KEGG level 3 pathways for the Chinese monal in different seasons. Table S6: Predicted functions of KEGG level 3 pathways for the Chinese monal at different ages.
