# Supplementary figures and images for: Seasonal Changes and Age-Related Effects on the Intestinal Microbiota of Captive Chinese Monals (Lophophorus lhuysii)
Source: Animals (Basel). 2024 Nov 26;14(23):3418. doi: 10.3390/ani14233418 (PMC11640307; doi:10.3390/ani14233418)

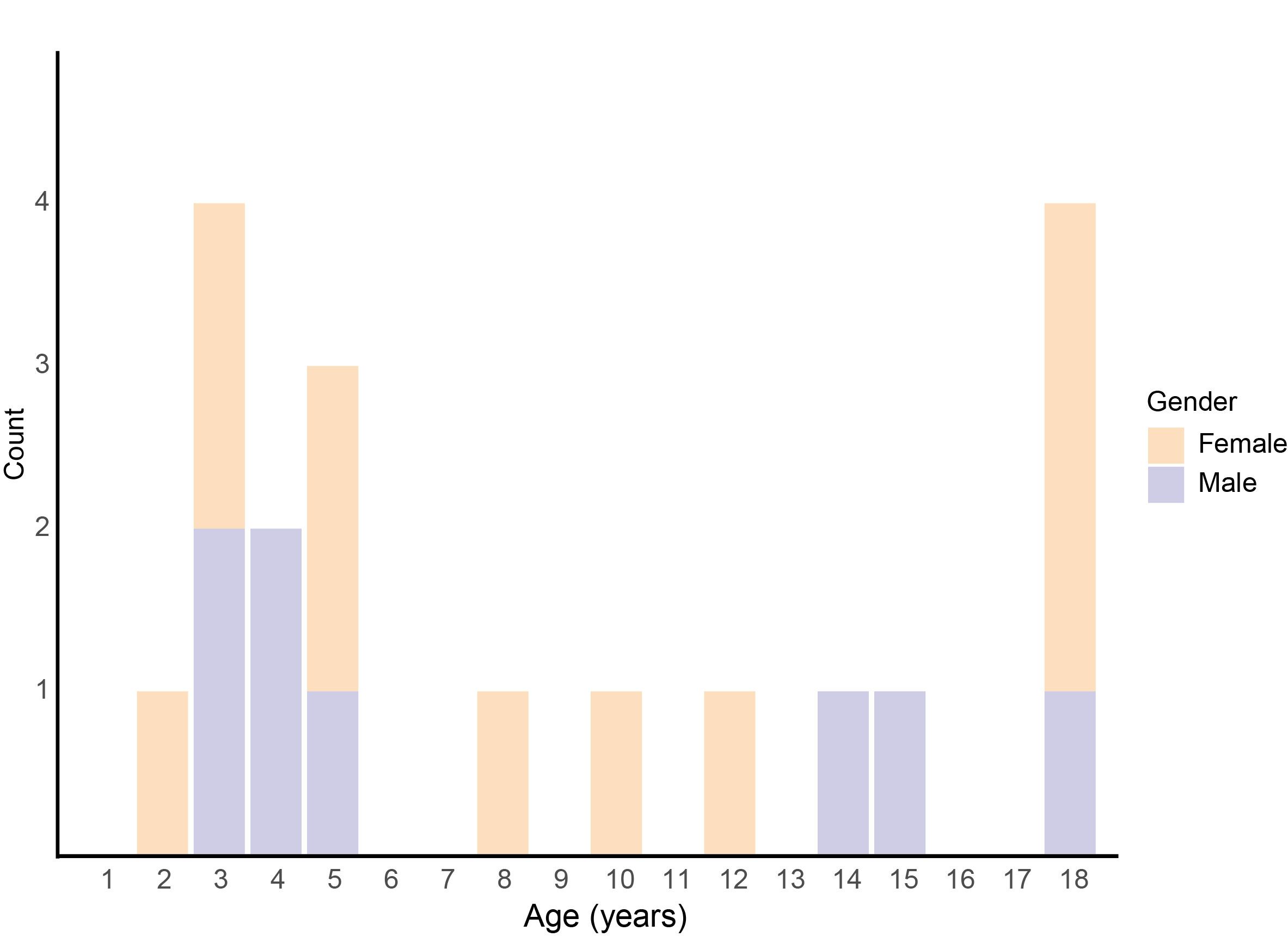

Supplement: Supplementary file 1 [file animals-14-03418-s001.zip › Supplementary Figure S1.jpg]

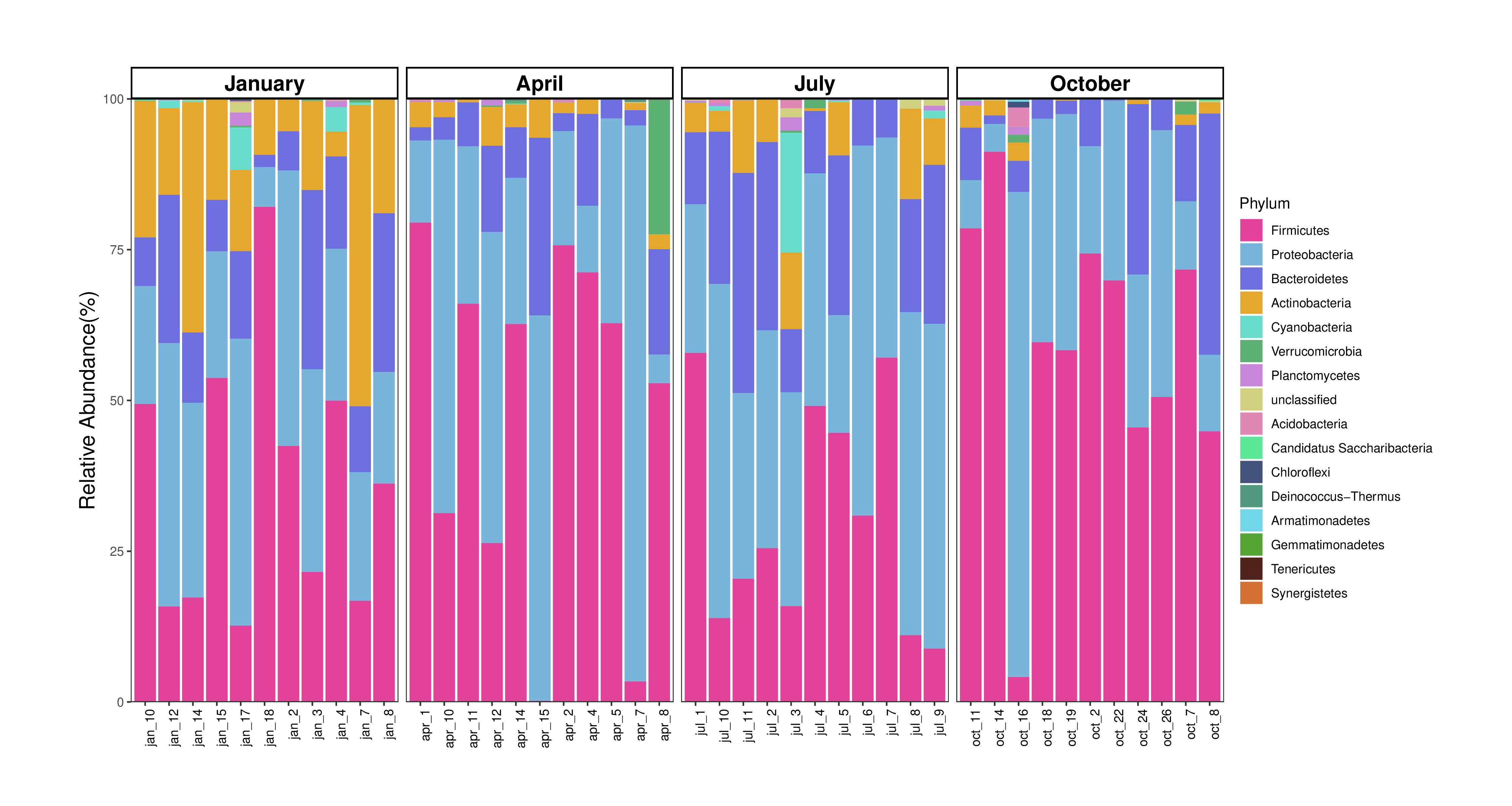

Supplement: Supplementary file 1 [file animals-14-03418-s001.zip › Supplementary Figure S2.jpg]

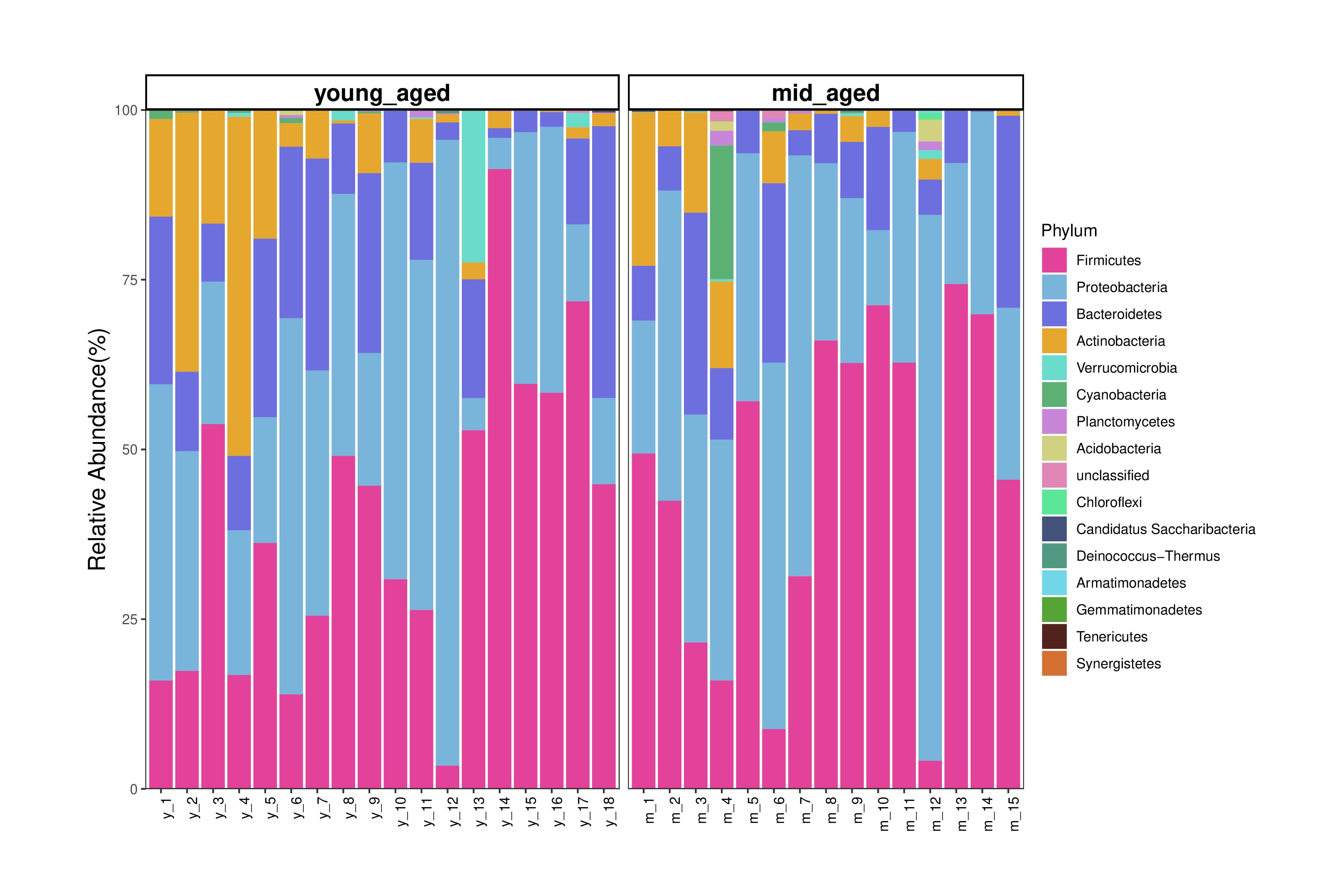

Supplement: Supplementary file 1 [file animals-14-03418-s001.zip › Supplementary Figure S3.jpg]

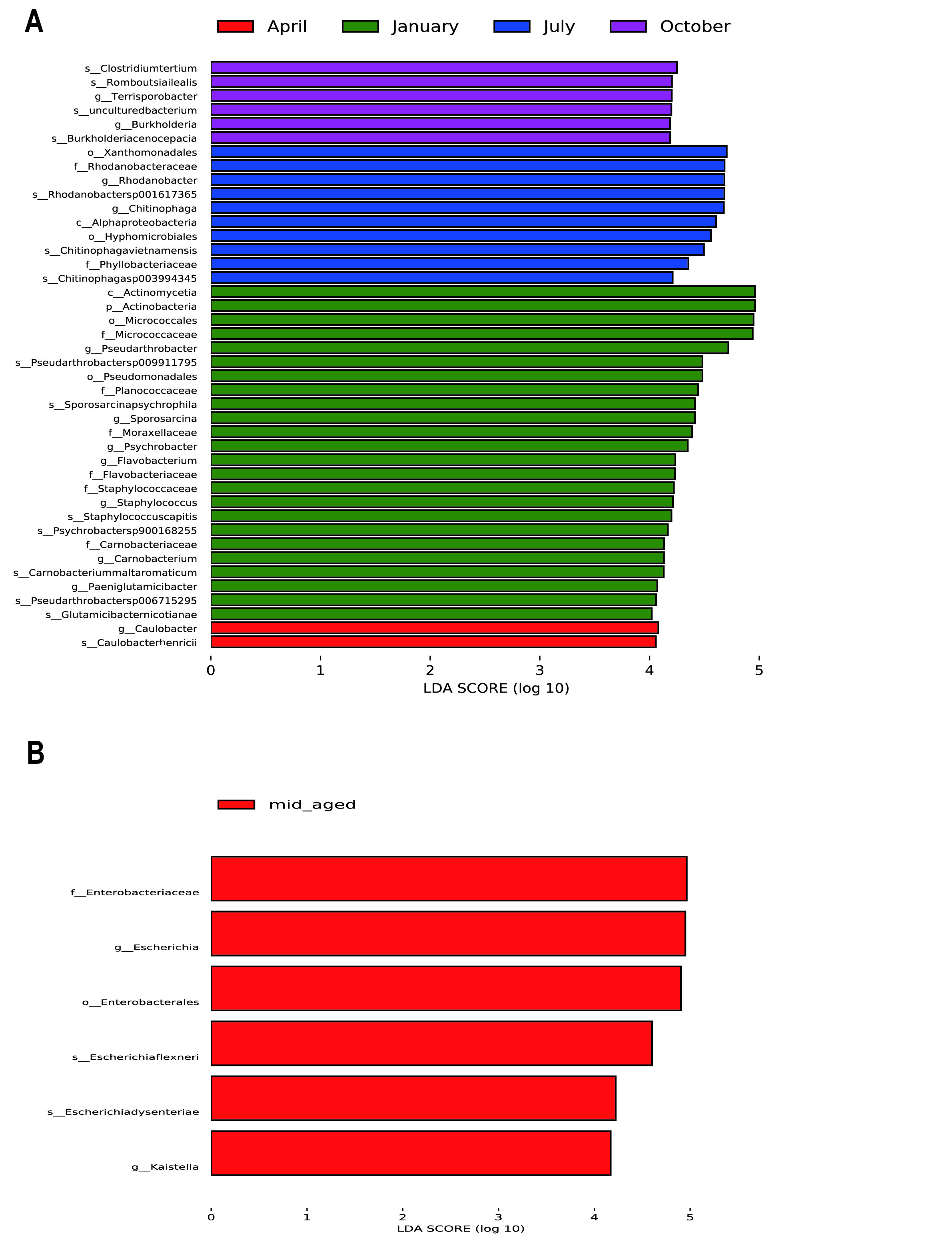

Supplement: Supplementary file 1 [file animals-14-03418-s001.zip › Supplementary Figure S4.jpg]
